# Improving ETa Estimation for *Cucurbita moschata* Using Remote Sensing-Based FAO-56 Crop Coefficients in the Lis Valley, Portugal

**DOI:** 10.3390/plants14213343

**Published:** 2025-10-31

**Authors:** Susana Ferreira, Juan Manuel Sánchez, José Manuel Gonçalves, Rui Eugénio, Henrique Damásio

**Affiliations:** 1 Instituto de Desarrollo Regional, UCLM Universidad de Castilla-La Mancha, 02071 Albacete, Spain; juanmanuel.sanchez@uclm.es; 2 IPC Instituto Politécnico de Coimbra, Escola Superior Agrária de Coimbra, CERNAS—Research Center for Natural Resources, Environment and Society, 3045-601 Coimbra, Portugal; jmmg@esac.pt; 3 ARBVL Associação de Regantes e Beneficiários do Vale do Lis, Quinta do Picoto, 2425-492 Leiria, Portugal; eugenio-rui@sapo.pt (R.E.); hdamasio71@gmail.com (H.D.)

**Keywords:** crop productivity, evapotranspiration, irrigation, soil moisture, sustainable agriculture, vegetation indices, water productivity

## Abstract

Efficient water management is essential for optimizing agricultural productivity in water-scarce regions such as the Lis Valley, Portugal. In situ measurements of soil moisture content (SMC) and electrical conductivity (EC), together with Sentinel-2-derived vegetation indices, were used to assess the crop water status and evapotranspiration dynamics of pumpkin (*Cucurbita moschata* ‘Butternut’) during the 2020 growing season. SMC and EC were measured at depths of 10, 20, 30, 50, and 70 cm using a TDR sensor, with strong correlations observed in the upper layers, indicating that EC can complement direct SMC measurements in characterizing near-surface moisture conditions. Sentinel-2 imagery was acquired to compute NDVI, SAVI, EVI, and GCI. In addition, NDVI values obtained from both a GreenSeeker^®^ sensor and Sentinel-2 imagery were compared, showing a similar temporal pattern during the season. By replacing the standard FAO-56 K_c_ values with those derived from each vegetation index, ET_a_ was recalculated to incorporate actual crop condition variability detected via satellite. ET_a_ estimates from RS-assisted vegetation indices agreed with those obtained using the FAO-56 method; independent ET_a_ measurements were not available for validation. Although such agreement is partly expected due to calibration, its confirmation for *Cucurbita moschata* under Mediterranean conditions—where published references are scarce—reinforces the method’s practical applicability for water management in data-limited settings. Water Productivity (WP) was estimated as 8.32 kg m^−3^, and Water Use Efficiency (WUE FAO-56) was calculated as 0.64 kg m^−3^, indicating high water use efficiency under Mediterranean smallholder irrigation conditions. These findings demonstrate that integrating high-resolution RS with continuous soil moisture monitoring can enhance precision irrigation strategies, increase crop yields, and conserve water resources in the Lis Valley.

## 1. Introduction

Pumpkins (*Cucurbita* spp.) are among the most extensively cultivated horticultural crops worldwide and are valued for their nutritional content and culinary versatility. Among the five principal species—*C. pepo*, *C. maxima*, *C. moschata*, *C. ficifolia* and *C. argyrosperma*—*C. moschata* ‘Butternut’ has emerged as a market leader due to its high yield potential and abundance of bioactive compounds [1]. In Portugal, this cultivar dominates spring–summer production, particularly in the central-western maritime zone (e.g., Lourinhã), thriving in well-drained soils. It remains sensitive to frost, waterlogging, and nutrient imbalances [2,3]. According to national statistics, the pumpkin cropping area reached 3583 ha in 2022, producing 97,960 t [4]. Despite promotional efforts such as the “Love Butternut” campaign, approximately 90% of production is exported, chiefly to the United Kingdom, France and Spain [5], highlighting the economic importance of this crop for local farmers and the regional horticultural sector.

Water availability is a critical factor in the productivity of *C. moschata*, particularly during the fruit set and filling stages, when water deficits significantly affect marketable yield [6,7]. In Mediterranean climates—characterized by mild, wet winters and hot, dry summers—efficient irrigation is essential to meet crop water requirements while ensuring sustainability. The Lis Valley Irrigation District (LVID), located on Portugal’s central-western coast, covers approximately 2000 ha and includes modern alluvial soils of high agricultural quality. However, farms are highly fragmented, with small parcels, and some areas face drainage challenges, which constrain irrigation modernization and sustainable agricultural development.

Remote sensing (RS) technologies have transformed irrigation monitoring by enabling spatially distributed, non-destructive assessments of crop status across temporal scales [8,9,10]. Satellite platforms such as Sentinel-2 provide free multispectral imagery at high spatial (10 m) and moderate temporal (5-day) resolution, enabling the derivation of vegetation indices (VIs) such as the Normalized Difference Vegetation Index (NDVI), Soil-Adjusted Vegetation Index (SAVI), Enhanced Vegetation Index (EVI), and Green Chlorophyll Index (GCI). These indices provide information on canopy vigor, photosynthetic activity, and water status, although their accuracy may be constrained by cloud cover and revisit frequency during key phenological events [11,12]. In contrast, proximal sensors such as the Trimble GreenSeeker^®^ allow frequent, in-field acquisition of VI data. They provide finer temporal resolution and enable real-time decision support in heterogeneous agricultural plots [13,14]. The combination of Sentinel-2 and GreenSeeker^®^ data has also been successfully applied to validate crop evapotranspiration estimation (ET_a_) in apple orchards [15].

Estimating crop coefficients (K_c_) using RS is a key step toward optimizing ET_a_ modeling and irrigation scheduling. The FAO-56 dual crop coefficient approach distinguishes between the basal crop coefficient (K_cb_), soil evaporation (K_e_), and water stress effects, although it typically requires local calibration [16]. Over recent years, robust correlations have been established between NDVI and K_c_ or K_cb_ for a variety of crops, including rice [17,18,19], wheat [20], maize [21], sorghum [22], grapevine [23], apple [15,24], and other horticultural or field crops [8,25,26,27,28,29].

Soil water balance models and dual K_c_-based approaches are increasingly applied to determine crop water and irrigation requirements and to improve irrigation scheduling in smallholder systems [30,31,32]. These approaches can also help reduce non-beneficial consumptive water use, particularly under arid and semi-arid conditions [32]. Furthermore, crop water management under limited or saline water requires careful ET_a_ estimation and irrigation planning, as salinity stress can significantly affect yield [33].

Despite these advances, few studies have simultaneously integrated satellite- and proximal-derived VIs for the calibration of FAO-56 dual crop coefficients in horticultural species such as *Cucurbita moschata*. Addressing this gap is crucial because *C. moschata* has high water requirements and significant economic importance, while smallholder farmers in Mediterranean systems need effective precision irrigation tools to improve yield and resource-use efficiency.

The main aim of this study is to improve the estimation of Kc and ETa for *Cucurbita moschata* by integrating both satellite (Sentinel-2) and proximal (GreenSeeker^®^) vegetation indices within the FAO-56 dual crop coefficient framework. Additionally, the study seeks to assess soil moisture dynamics and evaluate water use efficiency under representative irrigation practices in the Lis Valley, providing insights to promote more accurate field-scale water management and sustainable crop production.

## 2. Results

### 2.1. Meteorological Data

Meteorological variables were recorded during the *C. moschata* ‘Butternut’ growing season (6 June–13 September 2020). Table 1 presents the monthly mean values and totals for the study period. The average air temperature ranged from 17.9 °C in June to 20.7 °C in July, with a maximum of 30.7 °C recorded in September and a minimum of 23.2 °C in June. Wind speed varied between 1.3 and 1.8 m s^−1^, while cumulative precipitation during the season totaled 31 mm. Reference evapotranspiration (ET_o_) peaked in July, reflecting the combination of high temperatures and low precipitation. Although the growing season generally followed weather patterns typical of the Central Coast of Portugal, precipitation levels were lower than the historical averages for the year [34].

Heatwaves in July and September significantly influenced crop demand and the dynamics of the growing season, with 2020 being the hottest year since 1931 [34]. Relative humidity ranged from 70.5% in September to 84.0% in August. Solar radiation varied between 20.4 MJ m^−2^ day^−1^ in September and 25.3 MJ m^−2^ day^−1^ in July. Despite relatively high precipitation in August, most rainfall occurred on a single day, while consistently high temperatures maintained elevated evapotranspiration rates.

These meteorological conditions influenced *C. moschata* growth and development, affecting water requirements, biomass accumulation, and overall crop performance. The observed patterns provide context for irrigation management and the interpretation of soil moisture and crop evapotranspiration data in the following sections.

### 2.2. Soil and Water Dynamics

Water quality analysis conducted on 14 July 2020 indicated that the irrigation water was suitable for crop cultivation. The pH was slightly alkaline at 7.46, and the EC was 805.8 µS/cm, reflecting moderate salinity. Conductivity (C) measurements supported this classification at 832.4 µS/cm. Dissolved oxygen (DO) was high at 90.79 mg/L, favoring root respiration. Water temperature was 25.8 °C. Total dissolved solids (TDS) were low at 0.40 ppm, salinity (S) measured 0.52 PSU, and resistivity (R) was 1151.3 Ω·cm. All the values fell within acceptable thresholds for irrigation.

In addition to providing baseline water quality, continuous in situ measurements of SMC and EC offer agronomic context, supporting the interpretation of crop water status and observed evapotranspiration patterns. Although not derived from RS, these data are essential for understanding soil–plant interactions under field conditions.

#### 2.2.1. Variation on the Shallow Groundwater Table

**Figure 1:**
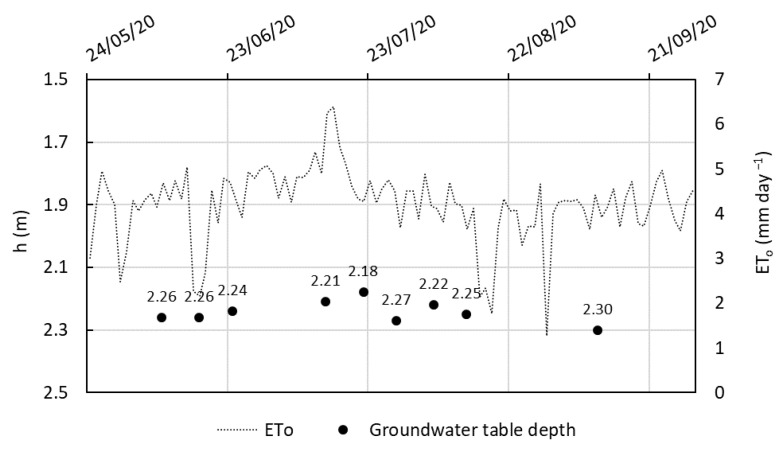
Groundwater table depth (meters below ground surface) measured throughout the 2020 growing season of *C. moschata* ‘Butternut’. ET_o_ (mm/day^−1^) is plotted as a continuous line. Data sources: field observations and local weather station.

The groundwater table depth was monitored throughout the 2020 growing season (Figure 1). In June, the water table was approximately 2.26 m below the soil surface and reached its shallowest point of 2.18 m on 22 July. Subsequently, it gradually deepened, reaching 2.27 m on 29 July and 2.30 m by the end of the season.

These fluctuations corresponded to prevailing weather conditions. From mid-June to late July, rainfall was minimal, and evapotranspiration frequently exceeded 4 mm/day, peaking above 6 mm/day in mid-July. High crop water uptake combined with soil evaporation likely contributed to the observed decline in groundwater levels.

#### 2.2.2. SMC and EC

**Figure 2:**
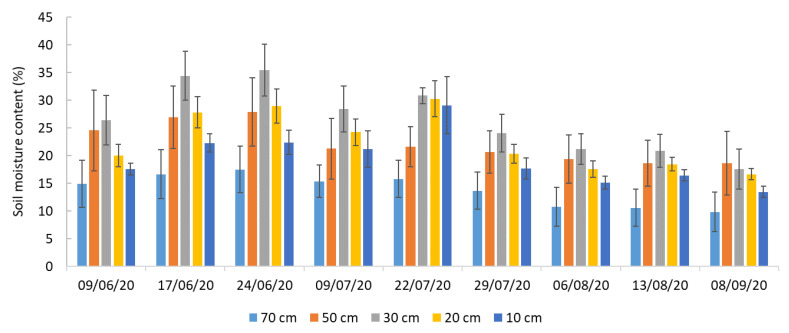
Volumetric soil moisture content (SMC, %) measured at 10, 20, 30, 50, and 70 cm during the 2020 *C. moschata* ‘Butternut’ growing season. Error bars represent standard deviation.

SMC and EC showed dynamic patterns throughout the 2020 growing season. As illustrated in Figure 2, SMC generally decreased from early June to early September, reflecting progressive soil drying under warmer summer conditions. Among the monitored depths, the 30 cm layer consistently retained the highest SMC, reaching 35% on 24 June. In contrast, the lowest SMC values were recorded at 70 cm (10% on 8 September) and 10 cm (13% on the same date). These results indicate that soil moisture was more readily available at intermediate depths, particularly around 30 cm, while the uppermost and deepest layers were more prone to water loss due to surface evaporation and limited deep infiltration.

EC followed a similar temporal pattern to that of SMC, peaking mid-season and declining toward the end of the growing season (Table 2). The highest EC values occurred at 30 cm, averaging 3.07 dS m^−1^, suggesting potential solute accumulation in this layer, likely driven by capillary rise and evaporative concentration. Deeper layers, such as 70 cm, exhibited lower EC values (average 1.90 dS m^−1^), reflecting reduced accumulation at greater soil depths.

Table 3 presents the regression results of SMC against EC across different soil depths. Strong correlations were observed in the upper layers: 10 cm (R^2^ = 0.89), 20 cm (R^2^ = 0.88), and 30 cm (R^2^ = 0.84). These findings indicate a strong coupling between soil moisture and salinity in the upper 30 cm.

In contrast, the correlations were negligible at deeper layers: 50 cm (R^2^ = 0.02) and 70 cm (R^2^ = 0.00). The ANOVA results confirmed statistical significance in the upper layers (*p* < 0.05), with F-values of 54.86 at 10 cm, 52.03 at 20 cm, 36.28 at 30 cm, whereas no significant relationships were found at 50 and 70 cm (*p* > 0.05).

#### 2.2.3. Soil Available Water Content and Storage

**Figure 3:**
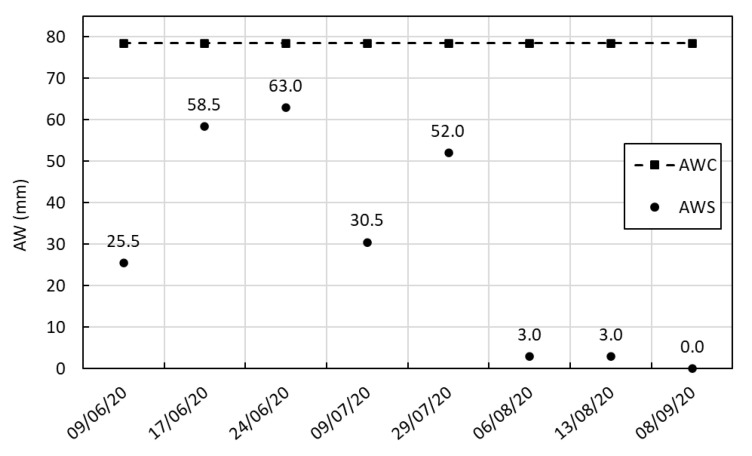
Observed total AWC and AWS of the soil during the 2020 growing season.

AWC was calculated for each soil layer based on field and permanent wilting point (Figure 3). The total AWC of the soil profile was estimated at 78.5 mm. AWS varied substantially throughout the 2020 growing season of *C. moschata* ‘Butternut’, reflecting both climatic conditions and irrigation management.

At the beginning of the monitoring period (9 June), AWS was relatively low (25.5 mm), indicating partial depletion of soil water reserves. It increased markedly by 17 June (58.5 mm), reaching a peak on 24 June (63 mm), likely due to rainfall or supplemental irrigation. By 9 July, AWS declined again to 30.5 mm, reflecting intensified plant water uptake and elevated evaporative demand. A partial recovery was recorded on 29 July (52 mm), followed by a sharp decrease to only 3 mm on 6 and 13 August.

After 5 August, coinciding with the onset of the harvest period, no further irrigation was applied, in accordance with the crop’s physiological maturation stage. During this phase, water demand naturally decreases, and drier soil conditions are often desirable to promote fruit ripening and improve storage quality. By 8 September, AWS reached 0 mm, indicating the complete depletion of plant-available water in the soil.

These results highlight a deliberate shift in water management strategy during the final stages of the crop cycle. Adequate soil moisture was maintained during the key vegetative and reproductive phases. The sharp decline in AWS after early August reflects the combined effect of irrigation cessation, sustained evapotranspiration, and the crop’s reduced water requirements during maturation.

### 2.3. Crop Coefficients and Growing Period Stages

**Figure 4:**
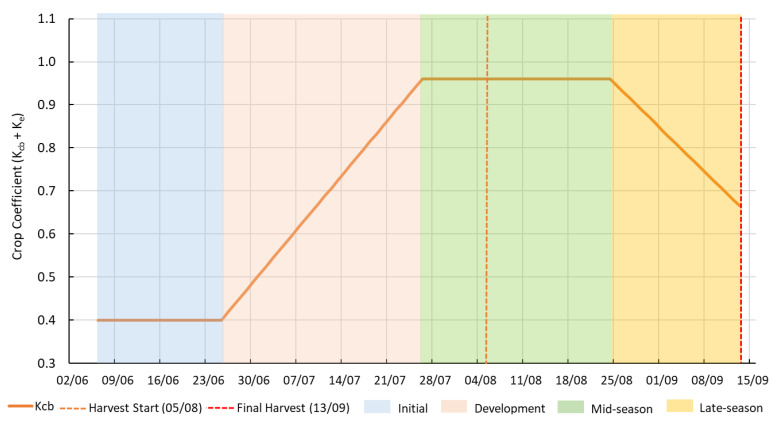
Crop coefficient (K_c_) curve for *C. moschata* ‘Butternut’ during the 2020 growing season (6 June–13 September), divided into four phenological stages: initial (blue), development (pink), mid-season (green), and late-season (orange). Vertical dashed lines indicate the onset of harvest (orange, on 5 August) and the final harvest (red, 13 September).

Figure 4 shows the K_c_ curve for *C. moschata* ‘Butternut’ during the 2020 growing season, which was adapted from the FAO-56 guidelines [16]. The four phenological stages —initial, development, mid-season, and late-season stages—lasted 20, 30, 30, and 20 days, respectively, as established in FAO Table 11 for pumpkins. Field observations in the experimental plot confirmed these stage durations.

The K_c_ values for each growth stage, derived from Tables 17 and 18 of FAO-56, were as follows: 0.4 for the initial stage, 0.96 for the mid-season stage, and 0.65 for the late-season stage. These coefficients reflect changes in crop water requirements across the growth cycle. The mid-season stage, coinciding with peak canopy development, showed the highest K_c_ value, indicating maximum evapotranspiration demand.

### 2.4. Evapotranspiration and Crop Water Use

Table 4 summarizes the water inputs from irrigation and precipitation during the 2020 *C. moschata* ‘Butternut’ growing season. Precipitation varied across the season, peaking at 23.0 mm during the vegetative growth phase (Phase III), with no precipitation recorded in the final phase (Phase IV). Irrigation was applied continuously, totaling 450 mm over the season. Stage-specific irrigation volumes were not measured; the total amount applied reflects typical farmer practices and provides a reliable basis for assessing seasonal crop water use.

ET_o_ ranged from 3.8 mm/day in Phase III to 4.8 mm/day in Phase II, with a cumulative seasonal value of 420.5 mm. ET_a_ followed a similar trend, totaling 307.3 mm, with the highest ET_a_ values observed during Phases II and III, corresponding to vegetative growth and fruit development.

These results underscore the crop’s water requirements and highlight the combined influence of precipitation and irrigation in meeting evapotranspiration demands. The highest water demands occurred during the vegetative and fruit development stages.

**Figure 5:**
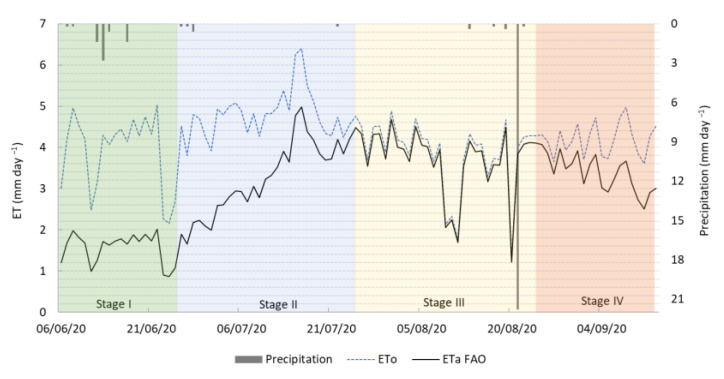
Daily ET_o_ and ET_a_ values calculated during the *C. moschata* ‘Butternut’ 2020 growing season. Precipitation is represented by vertical bars. The crop growth stages are distinguished by different colors.

Figure 5 illustrates the daily values of ET_o_, ET_a_, and precipitation throughout the growing season.

Notable differences between ET_o_ and ET_a_ were observed over the crop cycle, particularly when grouped by phenological phases. In Phase I (from 6 to 25 June), the crop exhibited the lowest water consumption relative to atmospheric demand, with an ET_a_/ET_o_ ratio of only 0.42. This was expected due to limited canopy development and low transpiration rates. At this stage, the crop had not yet established full ground cover, and a substantial portion of the ET_o_ likely corresponded to direct soil evaporation rather than transpiration.

ET_a_ increased sharply in Phase II (from 26 June to 25 July), resulting in a higher ET_a_/ET_o_ ratio of 0.80. This phase coincided with rapid canopy expansion and more efficient water use by the crop, as transpiration became the dominant component of evapotranspiration. The increase in ET_a_ indicates that the crop was approaching its peak water demand and optimizing the use of the available soil moisture.

Phase III (from 26 July to 24 August) marked the highest evapotranspiration efficiency, with an ET_a_/ET_o_ ratio of 0.88. This suggests that the crop was effectively meeting most of the atmospheric water demand, consistent with the intense metabolic activity and physiological needs during fruit development. The small gap between ET_o_ and ET_a_ at this stage reflects optimal growing conditions and sufficient water availability.

In Phase IV (from 25 August to 13 September), although ET_o_ remained relatively high, ET_a_ decreased, leading to a lower ET_a_/ET_o_ ratio of 0.68. This reduction likely reflects the natural decline in transpiration as the crop approached physiological maturity. ET_a_ values were calculated using nominal FAO-56 K_c_ and thus do not directly account for specific local irrigation decisions.

### 2.5. Satellite-Derived Vegetation Indices During the Growing Season

**Figure 6:**
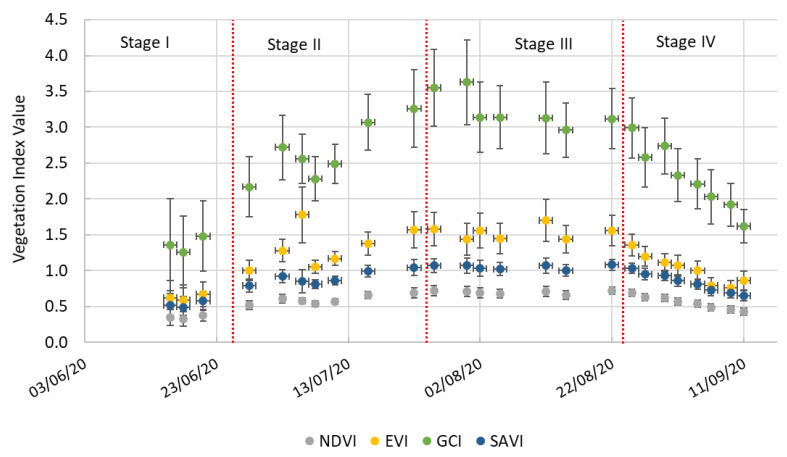
Temporal evolution of vegetation indices (NDVI, SAVI, EVI, and GCI) derived from Sentinel-2 imagery throughout the *C. moschata* ‘Butternut’ growing season. Error bars indicate the variability in the measurements, represented by the standard deviation. The crop growth stages are separated by red dashed lines.

The analysis of the vegetation indices (NDVI, SAVI, EVI, and GCI) revealed clear seasonal patterns (Figure 6).

In Phase I, all the vegetation indices exhibited relatively low values, reflecting limited canopy development at the beginning of the season. NDVI ranged from 0.29 to 0.38, SAVI from 0.44 to 0.58, EVI from 0.50 to 0.67, and GCI from 1.20 to 1.48. These modest values are consistent with early crop growth, when vegetative cover is sparse and soil background still contributes significantly to the satellite signal.

In Phase II, all the vegetation indices increased steadily and markedly, indicating vigorous vegetative growth and canopy expansion. NDVI values climbed to 0.69, while SAVI reached 1.04. EVI showed a strong upward trend, peaking at 1.57, and GCI increased sharply, surpassing 3.26 by late July. These trends reflect an active photosynthetic canopy with high chlorophyll content and biomass accumulation, corresponding to peak vegetative development.

During Phase III, all the indices remained high, confirming the fully developed canopy during the fruit maturation stage. NDVI remained stable around 0.68–0.72, while SAVI fluctuated between 1.00 and 1.08. EVI values ranged from 1.44 to 1.70, suggesting sustained transpiration and metabolic activity. GCI remained consistently high (3.12–3.63), indicating continued chlorophyll presence and healthy canopy conditions.

In Phase IV, a progressive decline was observed in all the indices, indicating senescence and reduced vegetative activity as the crop approached maturity and harvest. NDVI decreased from 0.69 to 0.43, SAVI from 1.03 to 0.65, and EVI from 1.36 to 0.86. GCI also dropped from 2.85 to 1.62. This decline is expected as the crop reduces its photosynthetic capacity and begins to dry down, accompanied by lower chlorophyll content and canopy density.

### 2.6. Comparison Between Proximal (GreenSeeker^®^) and Satellite-Derived NDVI

The NDVI values obtained from GreenSeeker^®^ and Sentinel-2 were highly consistent during the early and mid-growing season (June to late July 2020), with minimal discrepancies (mean absolute differences ~0.03). For example, on 22 July and 29 July, both sensors reported NDVI of 0.69 and 0.71, respectively.

From early August onward—coinciding with full canopy closure—Sentinel-2 NDVI tended to exceed GreenSeeker^®^ readings, likely due to differences in sensor geometry and spatial resolution under dense canopy conditions.

**Figure 7:**
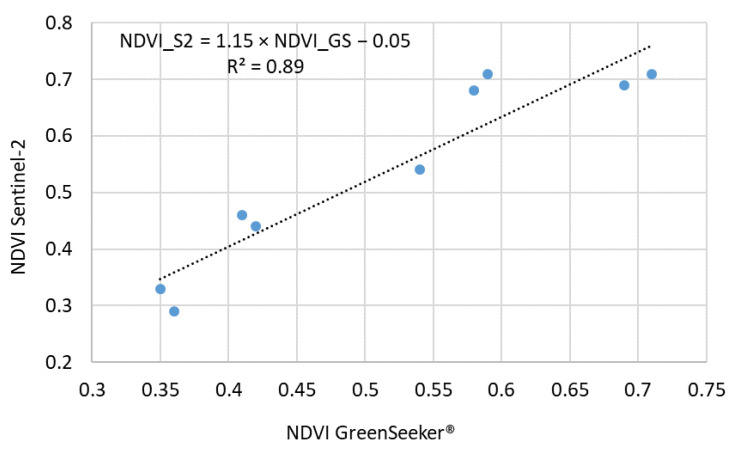
Correlation between NDVI (GreenSeeker^®^) and Sentinel-2.

A linear regression analysis between GreenSeeker^®^ and Sentinel-2 yielded R^2^ = 0.89 (*p* < 0.001), confirming a strong correlation (Figure 7). Despite a limited sample size (*n* = 9), proximal sensing reliably monitored early and mid-season canopy development.

This comparison highlights the complementary roles of satellite and proximal sensors: satellites support large-scale periodic monitoring, while proximal sensors provide high-resolution plot-level data. Divergences during high-biomass stages are important for irrigation management.

### 2.7. Calibration of the RS-Assisted K_c_-VI Relationship

A linear regression analysis revealed significant relationships between FAO-derived K_c_ values and all the tested vegetation indices (Table 5). NDVI and SAVI showed the strongest correlations across the full season (R^2^ ≈ 0.735, Multiple R = 0.857, *p* < 0.001), with low residual standard errors (0.112) and high F-values (>66, *p* < 0.001), indicating robust predictive performance. GCI also presented a strong correlation (Multiple R = 0.821, R^2^ = 0.674, *p* < 0.001), while EVI showed the weakest relationship (R^2^ = 0.500, *p* < 0.001), although still statistically significant.

Regression slopes (Coefficient of X_1_) were positive for all indices, with NDVI (1.393) and SAVI (0.930) showing the steepest gradients, confirming their suitability for K_c_ estimation.

The number of observations used in each regression was 26. Residuals were visually inspected and showed an approximately normal distribution, with no apparent pattern, supporting the validity of the linear regression assumptions.

The strength of the K_c_–VI relationships varied across crop growth stages (CGS) and their combinations (Table 6). For individual stages, Stage II and Stage IV exhibited the highest correlations for NDVI and SAVI (R^2^ = 0.692 − 0.863), whereas Stage I and III had insufficient data for reliable regression (N/A). Combined-stage analysis generally increased the robustness of the relationships. For example, NDVI and SAVI showed R^2^ values above 0.84 when combining Stages I + II + III or III + IV, while EVI remained the weakest index, particularly for early-stage combinations (R^2^ < 0.62). GCI displayed intermediate performance, with R^2^ ranging from 0.397 to 0.848, depending on the stage combination.

It is worth noting that regressions including fewer stages involved smaller sample sizes, which may reduce the robustness of the estimated relationships. Consequently, R^2^ values obtained from combined stages generally provide more reliable insights into K_c_–VI dynamics across the growing season, whereas regressions based on individual stages—particularly those with limited data—should be interpreted with caution.

The regression equations between each vegetation index (VI) and K_c_ are presented in Table 7. Each equation follows the linear form K_c_ = a × VI + b, with the slope (a) and intercept (b) fitted to the observed data. Model accuracy was assessed using the root mean square error (RMSE) and bias (BIAS), providing insight into the precision and potential systematic deviation of K_c_ estimates derived from each index.

NDVI and SAVI were the most accurate predictors, exhibiting the lowest RMSE and BIAS values, whereas EVI showed the largest deviations, and GCI provided moderate performance. These models allow for estimation of ET_a_ based on satellite-derived vegetation indices, reflecting temporal variability in canopy development and photosynthetic activity for *C. moschata* ‘Butternut.’

**Figure 8:**
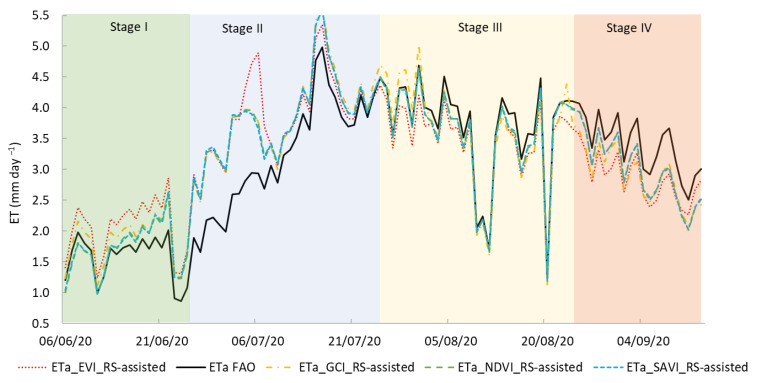
Comparison of ET_a_ based on the FAO model and vegetation index-Rs-assisted models (NDVI, SAVI, EVI, and GCI). The ET_a_ FAO is represented by a solid black line. The vegetation index-Rs-assisted ET_a_ values are shown as follows: EVI—red dotted line, GCI—orange dashed line, NDVI—green dashed line, and SAVI—blue dashed line. The crop growth stages are distinguished by different colors.

ET_a_ values derived from the FAO methodology were compared with those estimated from RS-assisted models based on NDVI, SAVI, GCI, and EVI (Figure 8). Across all the crop growth stages, RS-assisted methods reproduced the temporal dynamics of FAO ET_a_, with varying levels of agreement depending on the vegetation index.

During Stage I (initial growth), FAO ET_a_ ranged from 0.86 to 2.01 mm day^−1^. RS-assisted values were generally higher, particularly for EVI, which exceeded FAO ET_a_ by up to 0.40 mm day^−1^ on several days. NDVI and SAVI closely tracked FAO values, with small and consistent differences.

In Stage II (mid-season), ET_a_ increased substantially, with FAO estimates peaking at 4.98 mm day^−1^. RS-assisted approaches maintained strong agreement, although EVI and GCI produced larger differences during peak ET_a_ periods (early to mid-July), with EVI exceeding FAO ET_a_ by up to 0.91 mm day^−1^.

Stage III (late season) showed a gradual decline in ET_a_, with FAO values ranging from 1.22 to 4.69 mm day^−1^. RS-assisted models captured the seasonal decrease, with GCI showing the closest match, while EVI and NDVI tended to produce slightly higher values on low ET_a_ days.

In Stage IV (senescence), FAO ET_a_ decreased from 4.07 to 2.51 mm day^−1^. RS-assisted estimates tended to be higher, particularly on low ET_a_ days (e.g., 11 September, where EVI exceeded FAO ET_a_ by 0.25 mm day^−1^). Differences among NDVI, SAVI, and GCI were minimal in this stage.

Overall, all the RS-assisted models effectively reproduced the seasonal patterns of FAO ET_a_, with NDVI and SAVI showing the most consistent agreement, while EVI produced the largest deviations under peak ET_a_ conditions.

### 2.8. Crop Yield and Water Productivity

Table 8 summarizes the ET_a_ of *C. moschata* ‘Butternut’ estimated using the FAO-56 method and RS-assisted vegetation indices (NDVI, SAVI, GCI, and EVI), along with yield, WP, and WUE for the experimental season.

The ET_a_ values derived from RS-assisted indices were slightly higher than FAO-56 estimates across all indices. NDVI and SAVI resulted in similar increases in ET_a_, with values of 312.6 mm (2.9% higher) and 316.4 mm (3.0% higher), respectively. GCI produced the highest ET_a_ (318.0 mm, +3.5%), while EVI was slightly lower than GCI but still above FAO-56 (317.4 mm, +3.3%).

RS-assisted WUE followed a similar pattern to that of ET_a_, with GCI showing the highest WUE (0.661 kg·m^−3^, +3.3%), followed closely by EVI (0.660 kg·m^−3^, +3.2%), SAVI (0.658 kg·m^−3^, +2.8%), and NDVI (0.658 kg·m^−3^, +2.8%).

Overall, the RS-assisted indices provided slightly higher ET_a_ and WUE compared to the FAO-56 method, with GCI consistently producing the largest increases and NDVI the smallest.

## 3. Discussion

### 3.1. Water Table Fluctuations and Potential Irrigation Effects

The observed groundwater table fluctuations during the 2020 season closely followed the patterns of precipitation and evapotranspiration. The decline in groundwater depth from June to late July coincided with low rainfall and elevated ET_o_ (frequently > 4 mm day^−1^), reflecting the combined effect of increased crop water uptake and reduced aquifer recharge. Mechanistically, this aligns with the concept of hydraulic gradients driving soil–water fluxes: as ET_a_ exceeds local inputs, the shallow aquifer undergoes drawdown, a pattern commonly observed in Mediterranean regions with shallow, unconfined aquifers [35,36]. These dynamics are influenced by the interplay between soil hydraulic conductivity, aquifer connectivity, and crop water demand, highlighting the sensitivity of shallow aquifers to climatic variability.

A sustained recovery of the groundwater table during August–September is not evident in the monitoring well records. Although precipitation increased in August (Table 1) and ET_o_ declined from July peaks, the measured water-table depths after 22 July show a gradual deepening (2.27 m on 29 July; 2.30 m by season end), suggesting that any recharge that occurred was limited in magnitude or spatially heterogeneous and did not result in a net rise in the monitored piezometer. Transient shallowing around 22 July (minimum depth 2.18 m) likely reflects short-term soil–water dynamics and localized infiltration from minimal rainfall (0.2 mm on 21 July) rather than substantial aquifer recharge. These observations highlight the importance of accurate ET_a_ estimation to correctly interpret groundwater responses.

The absence of detailed irrigation logs (dates and event volumes) prevents precise quantification of irrigation impacts on groundwater. It is therefore plausible—although not evidenced by the available data—that on-farm supplemental irrigation during dry spells partially buffered soil moisture and local groundwater responses, as reported in similar Mediterranean contexts [37,38]. Consequently, inferences regarding irrigation effects must remain tentative. These observations underscore the value of integrated monitoring (continuous groundwater records, quantified irrigation schedules, and spatially distributed rainfall/soil moisture data) to distinguish climatic from anthropogenic drivers of shallow aquifer dynamics [39,40].

Recent advances in FAO-56 dual crop coefficient methods improve ET_a_ estimation by separating basal crop transpiration and soil evaporation, capturing crop growth effects and water stress [26,28,30]. Mechanistically, these approaches explain observed water table fluctuations under variable precipitation and irrigation scenarios, as ET_a_ can be more accurately partitioned into soil and crop components. Integration of local weather data, crop coefficients, and dual-K_c_ methods thus provides a robust framework for analyzing shallow aquifer dynamics in Mediterranean conditions, supporting better-informed irrigation and water resource management.

### 3.2. SMC and EC Dynamics

In the upper soil layers, SMC and EC exhibited a strong relationship (10–30 cm). The highest correlation occurred at 10 cm (R^2^ = 0.89, *p* < 0.001), with comparable associations at 20 cm (R^2^ = 0.88, *p* < 0.001) and 30 cm (R^2^ = 0.84, *p* < 0.001) (Table 3). These findings align with previous studies showing that EC is particularly responsive to moisture fluctuations in the upper profile, where evaporation, capillary rise, and biological activity are most active [41,42]. From a mechanistic viewpoint, this can be interpreted under the framework of FAO-56 dual crop coefficient approaches, in which upper-layer moisture responds rapidly to both evaporation and root water uptake, whereas deeper horizons are less affected [8,25,29].

At greater depths (50 and 70 cm), the SMC–EC relationship was negligible and statistically non-significant (R^2^ = 0.02 and 0.00; *p* = 0.75 and 0.91, respectively). Very low F-values and non-significant regression coefficients indicate that EC is not a reliable predictor of short-term moisture dynamics at these depths. This pattern is consistent with the notion that deeper soil horizons often have more buffered moisture regimes, which reduces the responsiveness of EC to near-surface hydrological variability [43,44]. Such behavior highlights the importance of understanding vertical soil hydraulic gradients and capillary flux limitations when interpreting EC signals across the profile.

Although EC correlates strongly with SMC in the upper 30 cm, methodological considerations apply. Sensor calibration, probe insertion variability, measurement frequency, and spatial heterogeneity in soil salinity may influence the transferability and precision of the SMC–EC relationships. Recent studies combining remote sensing vegetation indices with in situ SMC measurements have shown that integrating EC sensors with local ET_a_ estimation improves irrigation scheduling and soil water management [8,25,29,45]. Therefore, EC-based SMC estimations should be locally validated and, where feasible, complemented with direct moisture sensors, particularly for crops such as *Cucurbita moschata*, which extract water predominantly from the upper soil profile.

### 3.3. Growth Phases and Vegetation Index Dynamics

The analysis of vegetation indices (NDVI, SAVI, EVI, and GCI) during the growing season provided valuable insights into the physiological dynamics of *Cucurbita moschata* ‘Butternut’. The distinct growth phases observed in the crop exhibited specific patterns in vegetation index dynamics, indicative of physiological changes across the plant’s life cycle.

Phase I was characterized by low values for all vegetation indices, reflecting sparse canopy, limited photosynthetic activity, and reduced chlorophyll content [46,47,48]. NDVI ranged from 0.29 to 0.38, while SAVI, EVI, and GCI similarly indicated early-stage growth with strong soil background influence. These low ET_a_ values align with FAO-56 crop coefficient theory, where the K_cb_ is small during initial canopy development [26,27,28,29].

Phase II showed a marked increase in all vegetation indices, reflecting vigorous vegetative growth and canopy expansion. NDVI climbed to 0.69, and SAVI reached 1.04, indicating enhanced photosynthetic activity, canopy cover, and increasing ET_a_. In addition, VI and GCI increased, signaling high chlorophyll content and biomass accumulation [49,50]. These patterns align with FAO-56 mechanistic adjustments of K_cb_ according to canopy development, confirming that the crop’s water demand and transpiration rates rise rapidly as canopy density expands [26,27,28,29].

Phase III maintained high vegetation index values (NDVI 0.68–0.72; EVI 1.44–1.70; SAVI 1.00–1.08; GCI 3.12–3.63), reflecting full canopy development, sustained photosynthetic activity, and maximal biomass accumulation [51]. This corresponds to peak transpiration and water requirements, supporting the mechanistic interpretation of ETa based on VI-derived canopy dynamics combined with dual-K_c_ FAO-56 methods [8,25,29].

Phase IV exhibited a progressive decline in all indices (NDVI 0.43; SAVI 0.65; EVI 0.86; GCI 1.62), indicating senescence, reduced photosynthetic activity, and canopy drying as the crop approached maturity [52,53,54,55]. This decline aligns with mechanistic expectations of decreasing K_cb_ and ET_a,_ as canopy density and metabolic activity diminish.

Overall, these observations corroborate RS-based studies in similar Mediterranean horticultural systems [56,57,58] and support the use of vegetation indices to monitor ET_a_ and guide irrigation timing.

### 3.4. Calibration of K_c_–VI Relationships Across Growth Stages

Mechanistic calibration of K_c_–VI relationships links canopy development to K_cb_, enabling real-time adjustment of crop coefficients and precise irrigation aligned with peak transpiration periods (Phases II–III). This approach is essential for implementing precision or deficit irrigation strategies in water-scarce regions, allowing crop water requirements to be tailored to actual canopy development and improving the accuracy of ET_a_ estimates derived from satellite or proximal sensing data [8,25,26,27,28,29,59,60].

In the present study, NDVI and SAVI consistently demonstrated superior performance for estimating K_cb_, while EVI showed weaker correlations—likely due to saturation in dense canopies [61]. GCI exhibited moderate performance, capturing chlorophyll variations but showing greater variability depending on phenological stage and sample size [55]. From a mechanistic perspective, these differences can be explained by index sensitivity: NDVI and SAVI effectively capture fractional canopy cover, whereas EVI is prone to saturation under high LAI, and GCI reflects chlorophyll content with moderate structural influence.

In the context of the Lis Valley, phase-specific calibration enables irrigation events to be aligned with periods of peak transpiration, minimizing over-application and enhancing water productivity [62,63]. Given increasing pressure on regional water resources, the operational implementation of these tools can substantially improve decision-making for both large-scale operations and smallholder farmers. NDVI emerges as the most suitable vegetation index for practical and scalable irrigation management due to its robust performance across phenological stages, ease of interpretation, and strong correlation with crop coefficients—particularly during peak vegetative development. Its accessibility via multiple platforms, including SPIDERwebGIS© (http://maps.spiderwebgis.org/webgis/) (accessed on June and July 2025), Google Earth Engine (GEE), the Copernicus Open Access Hub, NASA Earthdata, and Sentinel Hub, facilitates efficient retrieval, processing, and analysis of multi-temporal imagery for precision irrigation and broader agro-environmental monitoring.

### 3.5. Implications of Sensor Geometry and Soil Background on NDVI Accuracy

The divergence observed between proximal sensing (i.e., near-surface measurements with the handheld GreenSeeker^®^ sensor) and satellite-based NDVI (Sentinel-2) values—particularly after full canopy closure—is consistent with limitations in sensor geometry and background interference reported in the RS literature [49,50]. In this study, GreenSeeker^®^ and Sentinel-2 NDVI values were largely consistent until the mid-season stage (late July), with a strong and statistically significant correlation (R^2^ = 0.89, *p* < 0.001), confirming the reliability of proximal sensing during early growth. However, from early August onward, GreenSeeker^®^ tended to underestimate NDVI compared to Sentinel-2 by approximately 0.15–0.18 units, likely due to sensor-specific saturation effects under dense canopy conditions.

As the canopy becomes denser, proximal sensors positioned at low altitudes (~0.5 m above the canopy) with narrow viewing angles primarily capture reflectance from the upper layer of sunlit leaves. This results in NDVI values dominated by vigorous photosynthetic surfaces with minimal soil influence [49,50]. In contrast, during late-season stages with high canopy closure, Sentinel-2 NDVI often exceeded GreenSeeker^®^ measurements. Differences in sensor footprint and signal integration explain this pattern: Sentinel-2 pixels (10 m) average reflectance over larger areas, including more uniform or less-stressed vegetation, whereas GreenSeeker^®^ captures high-resolution, plot-level variability, including shadows and canopy gaps [15,19]. Observation geometry also contributes, as GreenSeeker^®^ operates in nadir configuration under controlled lighting, while Sentinel-2 acquires data under varying solar angles and bidirectional reflectance distribution functions, introducing both atmospheric and geometric variability.

Empirical corrections or soil-adjusted indices, such as SAVI and EVI, can mitigate these inter-sensor discrepancies, improving alignment between proximal and satellite observations [49,50]. Combining both sources enhances irrigation management by providing high-resolution early- and mid-season monitoring from proximal sensors, alongside large-scale late-season assessments from satellite imagery [25,29].

From a practical perspective, understanding the geometric and spectral limitations of different sensors is crucial for end-users. In the LVID, proximal sensors such as GreenSeeker^®^ provide high-frequency, plot-level data but may be constrained in smallholder contexts due to labor and technical requirements. For large-scale monitoring or dense canopies, Sentinel-2 remains essential. Integrating both sources, potentially via data fusion, allows for comprehensive irrigation management tailored to heterogeneous field conditions while mechanistically linking observed canopy dynamics to ETa and crop water requirements.

### 3.6. Limitations and Final Considerations

It is worth noting that this study was conducted on a single field over one growing season, reflecting local *C. moschata* cultivation conditions. Due to the scarcity of large-scale pumpkin plots in the region, independent validation across multiple fields or years was not feasible. Nevertheless, the calibration results provide valuable insights into the K_c_–VI relationship for practical irrigation management under Mediterranean conditions. Climatic variability, soil heterogeneity, and management changes across years can influence crop performance and WUE; therefore, future research should focus on long-term monitoring to validate the stability of K_c_–VI relationships under varying environmental and operational conditions [64].

Additionally, the common practice of crop rotation on the farm complicates the year-to-year continuity of irrigation strategies and RS calibration for a specific crop like *C. moschata* ‘Butternut’. Although crop rotation enhances soil fertility and pest control, it contributes to variability in canopy structure, root distribution, and evapotranspiration dynamics. Future studies could incorporate rotational models and mixed cropping simulations to assess how water management protocols can be adapted or generalized across cropping systems [65,66].

Another limitation is the exclusive use of Sentinel-2 imagery and handheld sensors. Although effective, these tools may be spatially or temporally constrained under certain field conditions (e.g., cloud cover or limited revisit frequency). To overcome this, future trials should explore the integration of alternative platforms, such as UAV-mounted multispectral or hyperspectral cameras, which provide higher spatial resolution and customizable acquisition schedules. Comparative assessments across sensor types would improve scalability and accuracy in different operational contexts.

Importantly, direct measurements of ET_a_ were not feasible due to the lack of specialized field equipment (e.g., eddy covariance). Nevertheless, the comparison between FAO-56 and VI-K_c_ estimates provides a consistent indirect validation of the VI-based approach, supporting its practical use for irrigation management under Mediterranean conditions [8,25,26,27,28,29,30].

Despite favorable growing conditions and high yields during the trial, commercial cultivation of butternut squash was discontinued in subsequent seasons due to market constraints. The Portuguese domestic market remains underdeveloped, with most of the production destined for exports to the UK, France, and Spain. This reliance on external markets increases producers’ exposure to logistical disruptions and price volatility. To mitigate these vulnerabilities, policy incentives and investment in local supply chains and consumer awareness are needed to support market diversification [67].

Furthermore, challenges related to post-harvest handling and storage—particularly the lack of cooling and distribution infrastructure—undermine profitability. Integrating predictive tools from RS, such as vegetation indices to determine optimal harvest windows, could improve coordination between field production and market logistics [68].

Importantly, this study represented a unique opportunity to test precision irrigation in *C. moschata* under commercial conditions. Hitherto, very few farmers in the region have cultivated this crop for fresh markets, and there is limited scientific literature on RS-based crop coefficient modeling for *C. moschata*. Therefore, this study fills an important gap and lays the groundwork for future research in this area.

In summary, while this study confirms the potential of combining RS with soil moisture monitoring to enhance irrigation efficiency in *C. moschata* ‘Butternut’, broader implementation will require a more holistic approach. This includes not only refining technical aspects—through long-term and multi-platform validation—but also addressing economic sustainability, market access, and infrastructure needs. Future research should integrate agronomic innovation with supply chain resilience to support the long-term viability of precision irrigation in Mediterranean horticulture.

## 4. Materials and Methods

### 4.1. Description of the Study Site and Agronomic Management

**Figure 9:**
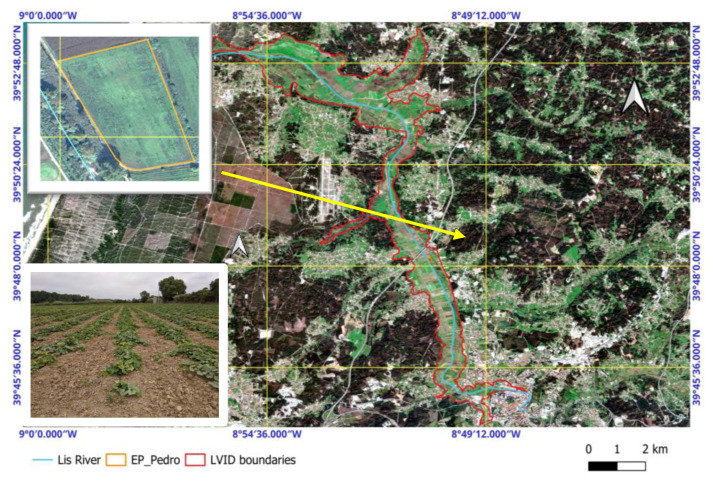
Satellite image of the Lis Valley region, acquired on 1 January 2025 via the Copernicus Browser (https://browser.dataspace.copernicus.eu/ (accessed on 30 April 2025)). The boundaries of the LVID are marked in red, and the Lis River is shown in blue. Orange lines delineate the plot boundaries. In the upper left-hand corner, the orange outline highlights the “Pedro Moteiro Experimental Plot,” extracted from Google Earth (https://earth.google.com (accessed on 30 April 2025)). In the lower left-hand corner, a photograph of the plot taken during the early growth stage in 2020 is shown. Following the Köppen–Geiger classification, the climate is Mediterranean semi-arid (Csb) [69], with mild winters and warm summers. The average annual precipitation is 790 mm, primarily occurring from October to March (Figure 10). The region has a mean annual temperature of 14.9 °C, with average maximum and minimum temperatures of 20.6 °C and 10.0 °C, respectively [70].

This study was conducted during the 2020 growing season in a plot of drip-irrigated butternut squash (*C. moschata* ‘Butternut’) located in the public irrigation district of LVID, (administrative headquarters: 39°51′22.1″ N, 8°50′56.1″ W), encompassing the municipalities of Leiria and Marinha Grande in the Central Region of Portugal (Figure 9). The LVID covers approximately 2000 ha of high-quality, modern alluvial soils. Certain areas, however, are affected by drainage constraints.

**Figure 10:**
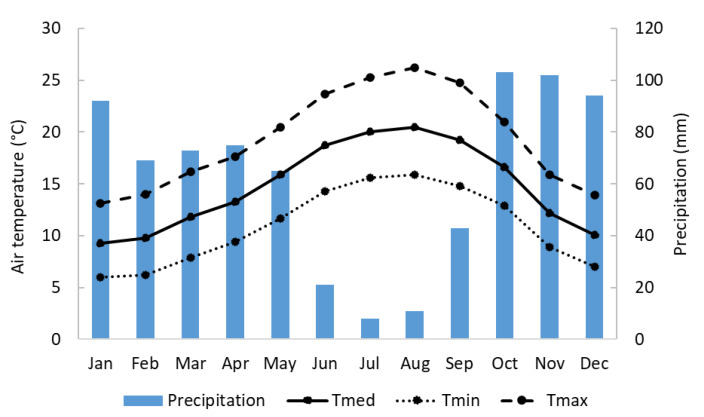
Average monthly air temperature and precipitation in the LVID (T_min_—minimum air temperature; T_med_—mean air temperature; T_max_—maximum air temperature) (adapted from [71]).

Horticultural crops, mainly cabbage, pepper, and tomato, occupy nearly 85 hectares of the irrigated area [72]. Their cultivation is projected to expand in the coming years. Horticultural expansion occurs mainly on smallholder family farms, where plots are typically smaller than one hectare and managed by aging farmers with limited market access. Production often serves subsistence needs, providing modest economic returns; however, these systems play an important socio-cultural role in the region [73].

**Figure 11:**
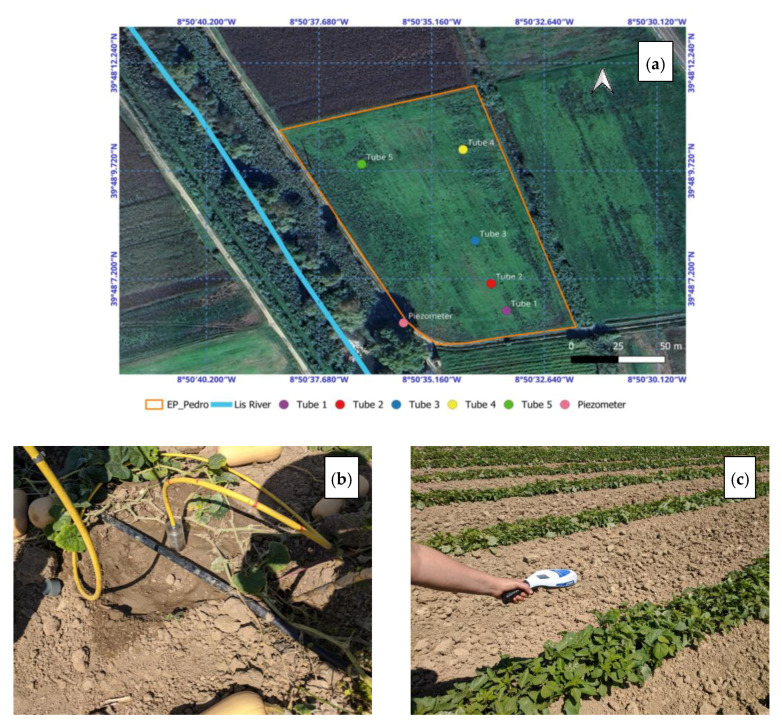
(**a**) Location of the five sampling points used for SMC, EC and NDVI measurements, as well as the piezometer position for groundwater monitoring; (**b**) Measurement of soil moisture using a TDR probe; (**c**) Use of the GreenSeeker^®^ handheld crop sensor to assess NDVI values in the field.

The experimental site, known as the “Pedro Moteiro Experimental Plot,” is located in Ortigosa (municipality of Leiria) and covers 1.7 ha (Figure 11). Historically, this plot has supported various horticultural crops: broccoli in 2018, pepper in 2019, and leek in 2021. Butternut squash was cultivated there for the first and only time in 2020, achieving a yield of 40 tons per hectare. This event marked the first commercial-scale production of butternut squash in the Lis Valley and provided a rare opportunity to collect empirical yield data under real farming conditions.

Data are available for only one season. Nonetheless, the case is noteworthy because the crop choice was exploratory and market-driven. The crop was discontinued in subsequent years due to logistical and economic constraints faced by the tenant farmer, including high labor demands, uncertain market returns, and the need for operational flexibility.

Sowing was carried out in alveolate trays under greenhouse conditions in mid-May, approximately three weeks prior to field transplanting. Temperature and humidity inside the greenhouse were controlled to ensure uniform germination and healthy seedling development. Transplanting was conducted on 6 June 2020, once the seedlings had developed 2–3 true leaves and were physiologically ready to adapt to field conditions.

The soil is medium-textured—composed of 49.1% coarse sand, 26.4% fine sand, 14.5% silt, and 10% clay—resulting in a sandy loam texture. It has a bulk density of 1.6 g cm^−3^, 0.85% organic matter, pH 7.4, and EC of 0.317 dS m^−1^, indicating no salinity concerns. Nutrient levels were adequate, with a Ca/Mg ratio of 3.0 and a Mg/K ratio of 18.2 (analyses conducted in 2020).

Irrigation was applied via a drip system, fed by water from the Vala do Carvão, occasionally supplemented with basin flooding. Irrigation scheduling was performed empirically, relying on visual crop indicators, soil moisture observations, and weather forecasts. While digital flow meters were not available, the estimated irrigation volumes provide a practical basis for calculating water productivity (WP) and water use efficiency (WUE) under typical smallholder conditions. Drainage was facilitated through a network of ditches that balanced aeration in the root zone with adequate soil moisture. Fertilizers were applied based on soil test recommendations.

Weed control was achieved through a combination of mechanical weeding between rows and targeted herbicide applications. This approach ensured the dominance of *C. moschata* and effectively controlled competing weeds, particularly *Datura stramonium*.

The main phytopathological threats included anthracnose (*Colletotrichum orbiculare*), downy mildew (*Pseudoperonospora cubensis*), and powdery mildew (*Erysiphe cichoracearum*). Preventive treatments were applied in periods of high disease risk to minimize potential crop losses.

Harvest was conducted manually using pruning shears, from 5 August to 13 September 2020, prioritizing fruits that were fully mature (golden-yellow color and shriveled peduncle). The total crop cycle lasted approximately 100 days.

The availability of irrigation and yield data enabled the calculation of water productivity (WP) and water use efficiency (WUE). WP (kg m^−3^) was calculated as the ratio of yield (kg ha^−1^) to the total amount of water applied (precipitation + irrigation, in m^3^ ha^−1^). WUE was calculated as the ratio of total actual evapotranspiration (ET_a_, m^3^ ha^−1^) to the total water applied. This approach follows the methodology used in [16].

### 4.2. Soil Moisture and Groundwater

SMC and EC were measured at depths of 10, 20, 30, 50, and 70 cm, using a portable Time Domain Reflectometry (TDR) probe (H2D model, IMKO; Figure 11b) inserted in permanently installed access tubes.

Measurements were taken at five representative locations (Figure 11a) over nine field campaigns between June and September 2020. Observations were made at approximately 15:00 h on the following dates: 9 June, 17 June, 24 June, 9 July, 22 July, 29 July, 6 August, 13 August, and 8 September. Mean values across the five locations were used to characterize soil moisture and EC trends throughout the season. EC was monitored as a general indicator of soil salinity, where elevated values can signal higher concentrations of soluble salts that may restrict plant water uptake.

The available water capacity (AWC) was estimated as the difference between volumetric field capacity (32%) and the wilting point, determined as 18%, 19%, 20%, and 21% at depths of 10, 20, 30, 50, and 70 cm, respectively. These values were estimated based on the soil type and texture, using the graphical data from Molina Jr. [74]; they provided insights into the water retention properties of the soils.

Soil water storage (AWS) was calculated by integrating the TDR-based temporal moisture readings. This allowed periods with adequate moisture or potential drought stress to be identified. AWS trends helped guide irrigation management during the season. The total AWC of the root zone was obtained by summing the contributions of all the layers. As this represents the effective rooting depth, the total AWC can be considered effectively constant throughout the season.

Groundwater levels were monitored using a piezometer installed at a depth of 3 m near the main observation transect (coordinates: 39°48′06.172″ N, 8°50′35.791″ W).

These measurements of soil moisture and groundwater dynamics provided a robust basis for evaluating soil water availability throughout the growing season and informed irrigation management. They also supported the interpretation and validation of crop evapotranspiration estimates derived from both the FAO-56 dual crop coefficient method and the NDVI-based remote sensing approach.

### 4.3. Agrometeorological Data and Crop Water Use Estimation (FAO-56)

Weather conditions during the growing season were monitored using an automated agrometeorological station located within the Lis Valley region, approximately 4 km from the experimental plot (N 39°51′22.32″//W 8°50′56.44″). The site was selected for its uniform topography and consistent weather patterns, ensuring representative climatic data. Sensors were mounted 2 m above the soil surface and data were recorded hourly. The station measured air temperature, relative humidity, wind speed, wind direction, incoming solar radiation, and precipitation, following standard protocols described in previous studies [75].

ET_a_ was estimated using the dual crop coefficient approach (FAO-56) [16]:

(1) $${ET}_{a}=K_{\mathrm{cb}}+K_{e}\cdot {ET}_{o}$$

where ET_a_ is the actual crop evapotranspiration (mm day^−1^), K_cb_ is the basal crop coefficient, K_e_ is the soil evaporation coefficient, and ET_o_ is the reference evapotranspiration (mm day^−1^).

Daily ET_o_ was computed using the FAO-56 Penman–Monteith equation for a reference grass surface [16]:

(2) $${ET}_{o}=\frac{0.408\cdot ∆\cdot \left(R_{n}-G\right)+\gamma \cdot \frac{900}{T+273}\cdot u_{2}\cdot (e_{s}-e_{a})}{\Delta +\gamma \cdot (1+0.34\cdot u_{2})}$$

where ET_o_ is the reference evapotranspiration (mm day^−1^), R_n_ is the net radiation at the crop surface (MJ m^−2^ d ^−1^), *G* is the soil heat flux density (MJ m^−2^ d ^−1^), T is mean daily air temperature at 2 m height (°C), u_2_ is the wind speed at 2 m height (m s^−1^), e_s_ is the saturation vapor pressure (kPa), e_a_ is the actual vapor pressure (kPa), Δ is the slope of the vapor pressure curve (kPa °C^−1^), and γ is the psychrometric constant (kPa °C^−1^).

The dual crop coefficient method was selected to estimate ET_a_ with greater accuracy, calculating plant transpiration separately from soil evaporation. This approach is particularly useful in conditions where transpiration and evaporation are differently influenced by environmental and management factors, such as in drip-irrigated systems, crops with incomplete canopy cover, and soils with varying surface wetness [76].

Following the FAO-56 guidelines, the K_cb_ was estimated based on the crops’ development stages, using reference values from Table 17 and adjusting them according to Table 18 [16]. The adopted values were K_cb_ini_ = 0.15 for the initial stage (nearly bare soil), K_cb_mid_ = 0.90 during mid-season (dense canopy, >80% ground cover), and K_cb_end_ = 0.65 for the late season (partial senescence, infrequent irrigation).

Daily K_cb_ values were linearly interpolated between these stages to account for the crops’ phenological development. The total crop coefficient (K_c_) was calculated as:

(3) $$K_{c}=\left(K_{\mathrm{cb}}+K_{e}\right)$$

The K_e_ was estimated based on the fraction of the soil surface that is both exposed and wetted, represented by the parameter ‘*few*’. The fraction of the ground shaded by the crop canopy (*fc*) was estimated from field observations, while the fraction of the soil surface wetted by irrigation (*fw*) was set to 0.3, following FAO-56 guidelines for subsurface drip irrigation. The exposed and wetted fraction of the soil surface (*few*) was then calculated as:

(4) $$few=min\left(1-fc,fw\right)$$

At the start of the cycle (early June), with minimal canopy cover (*fc* ≈ 0.0), *few* ≈ 0.3. By late July, as the canopy reached approximately 90% coverage (*fc* ≈ 0.90), *few* decreased to ≈0.10, reflecting a reduced potential for soil evaporation.

The soil evaporation coefficient was calculated using the simplified form of the dual approach:

(5) $$K_{e}=min\left(K_{c\_\max}-K_{\mathrm{cb}},few\cdot K_{c\_\max}\right)$$

where K_c_max_ represents the maximum crop coefficient following rain or irrigation events. In the present study, K_c_max_ was set to 1.20, a typical value for many horticultural crops. This formulation ensures that K_e_ is reduced as canopy cover increases and/or soil wetness decreases, dynamically reflecting the conditions that limit soil evaporation.

The dual crop coefficient approach provided a more realistic simulation of evapotranspiration throughout the crop cycle, accounting for the dynamic contributions of soil evaporation and crop transpiration. This method is especially relevant for crops grown in fields with partial shading and localized irrigation, where the evaporation and transpiration processes differ substantially [77].

The growth stages for butternut squash—initial, development, mid-season, and late season—were defined based on Table 11 of the FAO-56 guidelines, with a total growth period of 100 days [16].

It is worth underlining that the transplantation date was selected for calculating K_c_ini_ rather than the sowing date, as it represents the point at which the crop begins to interact with the fields’ environmental conditions. While sowing occurs in a greenhouse, where environmental factors such as temperature and solar radiation are controlled, transplantation marks the transition to field conditions, where the crop is exposed to fluctuating weather conditions and changing water demands. As the plant establishes itself in the soil and adapts to its new environment, its water requirements evolve. Therefore, using the transplantation date for calculating K_c_ini_ provides more accurate evapotranspiration estimates, thereby more effectively reflecting field conditions. This approach is consistent with standard agricultural practices [16].

### 4.4. Satellite Image Acquisition and Pre-Processing

Satellite imagery was acquired from Sentinel-2 Level-2A products, downloaded from the Copernicus Data Space Ecosystem (https://browser.dataspace.copernicus.eu/ (accessed on 30 April 2025)). These products provide Bottom-of-Atmosphere (BOA) reflectance values—i.e., surface reflectance corrected for atmospheric effects—processed using the ESA Sen2Cor algorithm. The atmospheric corrections include adjustments for aerosols, water vapor, and cirrus clouds, based on the 6S radiative transfer model [78].

A total of 26 cloud-free scenes (≤5% cloud cover), covering the period from transplantation to harvest, were selected for analysis. The Level-2A products do not include cross-sensor harmonization between the Sentinel-2A and Sentinel-2B satellites, which may lead to inter-sensor variability. To reduce these differences, empirical radiometric correction factors were applied, together with the use of soil-adjusted vegetation indices—such as SAVI and EVI—as recommended in previous studies [48,49].

The vegetation indices analyzed in this study included NDVI, SAVI, EVI, and GCI. Their computation used the 10 m resolution bands B2 (Blue, 490 nm), B3 (Green, 560 nm), B4 (Red, 665 nm), and B8 (Near-Infrared, 842 nm). These bands capture reflectance in the visible and near-infrared spectrum, providing sensitivity to chlorophyll content and canopy structure, which are critical for vegetation monitoring.

Individual bands were exported as 16-bit GeoTIFFs in QGIS (v3.42.1, Münster) using the ‘Export Raster’ function and consequently clipped to the experimental plot boundary using a shapefile. Table 9 summarizes the number of cloud-free Sentinel-2 scenes available for each phenological stage of *C. moschata* ‘Butternut’.

### 4.5. Vegetation Index Calculation

To monitor vegetation health and productivity over the growing season, four complementary vegetation indices were calculated using the QGIS Raster Calculator. The overall objective was to assess crop performance under different environmental conditions and identify the index most suitable for the K_c_ (VI) calibration equation [79]. These indices were selected because they jointly capture chlorophyll content, minimize soil-background effects, provide sensitivity across a range of biomass, and detect stress signals.

Vegetation indices quantify specific spectral reflectance properties, enabling the detection of variations in chlorophyll content, which is directly related to photosynthetic activity. Continuous monitoring throughout the growing season supports the optimization of management practices and improves yield predictions [80].

The NDVI is one of the most widely used indicators for vegetation monitoring, providing a reliable estimate of chlorophyll content and photosynthetic activity [46]:

(6) $$NDVI=\frac{\left(NIR-RED\right)}{\left(NIR+RED\right)}$$

where NIR is the Near-Infrared reflectance and RED is the Red reflectance.

The SAVI was designed to minimize the influence of soil background, particularly in areas with sparse vegetation cover [50]:

(7) $$SAVI=\frac{\left(NIR-RED\right)}{\left(NIR+RED+L_{S}\right)}\times (1+L_{S})$$

where NIR is the Near-Infrared reflectance, RED is the Red reflectance, and $L_{S}$ is the soil correction factor (0.5).

The EVI provides improved sensitivity to high-biomass regions and is less influenced by atmospheric conditions and canopy background noise [81]:

(8) $$EVI=G\times \frac{\left(NIR-RED\right)}{\left(NIR+C1\times RED-C2\times BLUE+L\right)}$$

where NIR is Near-infrared reflectance, RED is the Red reflectance, BLUE is the Blue reflectance, *G* = 2.5 (gain factor), *C*_1_ = 6, *C*_2_ = 7.5 (coefficients for the bands), and *L* = 10,000 (background adjustment).

The GCI is sensitive to chlorophyll concentration and serves as an effective indicator of photosynthetic activity [49]:

(9) $$GCI=\frac{NIR}{GREEN}-1$$

where NIR is the Near-Infrared reflectance and GREEN is the Green reflectance.

All raster layers were clipped to the experimental plot boundary using a shapefile (EPSG:4326). A spatial resolution of 10 m—corresponding to the native resolution of the Sentinel-2 bands used—was maintained for all bands, ensuring spatial consistency with field measurements.

For ground-based NDVI, a GreenSeeker^®^ handheld active optical sensor (Trimble, Sunnyvale, CA, USA) was used (Figure 11c). This sensor emits red (656 nm) and near-infrared (774 nm) light and measures canopy reflectance. Measurements were taken at approximately 15:00 h local time under stable lighting conditions, on the same dates as the SMC and EC measurements (see Section 2.2 and Figure 2). The sensor was held approximately 0.5 m above the canopy along the central axis of each point (near the five TDR tubes). Around each plant, measurements were made in four cardinal directions, forming a 1 m × 1 m grid, with continuous sampling and calculation of the mean NDVI per transect. Three replicates per point were collected to ensure reliability.

Combining ground-based and satellite-derived NDVI provides high spatial resolution from proximal sensing while maintaining the broader temporal coverage of satellite imagery [15,19]. This dual approach, mainly used for calibration, enhances the robustness of vegetation monitoring and provides a more comprehensive view of crop health, supporting better management and decision-making throughout the growing season.

### 4.6. Statistical Analysis

The relationship between SMC and EC at different soil depths (10–70 cm) was evaluated using linear regression, Pearson’s correlation, and ANOVA to explore depth-dependent interactions between moisture and salinity.

Linear regression models were applied to relate FAO-56 K_c_ to each vegetation index (NDVI, SAVI, GCI, and EVI), with model performance assessed via R^2^, residual standard error, slope (coefficient of X_1_), and ANOVA. These models enabled ET_a_ to be estimated from satellite-derived vegetation indices, incorporating temporal variability in canopy development.

Comparisons between proximal sensor (GreenSeeker^®^) NDVI and Sentinel-2–derived NDVI were performed using linear regression and Pearson’s correlation to evaluate consistency and potential deviations across the growing season.

Differences between RS-assisted ET_a_, WP, and WUE and FAO-56 reference values were calculated as relative percentage changes. Linear regression and ANOVA were used to assess the relationship between Kc derived from vegetation indices and ET_a_, WP, and WUE.

All the statistical analyses were performed using JASP v0.19.1 (University of Amsterdam, Amsterdam, The Netherlands); statistical significance was considered at *p* < 0.05.

## 5. Conclusions

The integration of Sentinel-2 imagery with proximal NDVI sensing provides a robust framework for the simulation of precision irrigation strategies for *C. moschata* ‘Butternut’ under Mediterranean conditions. Of the four vegetation indices tested, NDVI exhibited the strongest relationship with the dynamic K_c_ (R^2^ ≥ 0.73), enabling stage-specific irrigation scheduling in simulations. Simulated irrigation based on these relationships resulted in estimated high water use efficiency (WUE = 0.64 kg m^−3^) and water productivity (WP = 8.32 kg m^−3^), indicating the potential of this approach to reduce water consumption without compromising yield.

The combined use of drip irrigation, TDR sensors, and handheld NDVI measurements offers a low-cost, scalable solution suitable for smallholder farmers. By integrating meteorological inputs, soil moisture data, and high-resolution remote sensing, the proposed methodology supports real-time decision-making and responsive irrigation management.

Future multi-season trials are needed to validate the stability of K_c_–VI relationships across different climatic conditions and crop cycles. Such validations will be critical for the broader adoption of this integrated remote sensing-based strategy, promoting sustainable water management and resilient horticultural production in water-limited regions.

Overall, this study demonstrates the value of combining RS with ground-based measurements to refine crop water management and advance precision agriculture in Mediterranean environments.

## Figures and Tables

**Table 1:** Summary of monthly meteorological variables recorded during the 2020 *C. moschata* ‘Butternut’ growing season, from transplanting to final harvest.

Season Month	T_mean_ (°C)	T_max_ (°C)	RH_mean_ (%)	R_s_ (MJ m^−2^ day^−1^)	u_2_ (m s^−1^)	P (mm)	ET_o_ (mm day^−1^)
June ^1^	17.9	23.2	81.1	22.5	1.8	7.6	4.0
July	20.7	27.7	79.5	25.3	1.6	0.2	4.8
August	20.1	25.8	84.0	20.5	1.7	23	3.8
September ^2^	20.1	30.7	70.5	20.4	1.3	0.2	4.1
Average/Total	19.7	26.9	78.8	22.2	1.6	31	4.2

T_mean_ is the mean air temperature, T_max_ is the mean maximum air temperature, RH_mean_ is the mean relative humidity, R_s_ is the global solar radiation, u_2_ is the wind speed at 2 m height, P is the total monthly precipitation, and ET_o_ is the mean monthly reference evapotranspiration calculated using the FAO-56 PM equation. ^1^ Values correspond to measurements starting on 6 June; ^2^ Values correspond to measurements ending on 13 September.

**Table 2:** Electrical conductivity (EC, dS·m^−1^) at soil depths of 70, 50, 30, 20, and 10 cm. Values represent mean ± standard deviation. The last row shows seasonal averages.

Date	70 cm	50 cm	30 cm	20 cm	10 cm
9 June	1.8 ± 0.3	2.7 ± 0.5	2.9 ± 0.3	2.4 ± 0.2	2.1 ± 0.2
17 June	1.8 ± 0.3	2.7 ± 0.6	3.4 ± 0.2	2.7 ± 0.2	2.5 ± 0.1
24 June	1.9 ± 0.2	3.0 ± 0.5	3.5 ± 0.3	2.9 ± 0.3	2.6 ± 0.2
9 July	2.0 ± 0.2	2.7 ± 0.4	3.3 ± 0.3	2.7 ± 0.3	2.5 ± 0.2
22 July	1.9 ± 0.2	2.8 ± 0.4	3.2 ± 0.1	2.8 ± 0.2	2.7 ± 0.3
29 July	2.1 ± 0.2	3.0 ± 0.6	3.1 ± 0.2	2.4 ± 0.1	2.3 ± 0.2
6 August	1.9 ± 0.2	2.8 ± 0.2	2.6 ± 0.3	2.0 ± 0.3	1.9 ± 0.2
13 August	1.9 ± 0.3	2.8 ± 0.3	2.8 ± 0.1	2.2 ± 0.2	2.1 ± 0.2
8 September	1.8 ± 0.1	2.8 ± 0.5	2.8 ± 0.3	2.2 ± 0.2	1.8 ± 0.1
Average	1.90	2.79	3.07	2.48	2.28

**Table 3:** Correlations between SMC and EC at different depths. Bold values indicate statistically significant correlations (R^2^ > 0.7, *p* < 0.05).

Pair of Variables	Multiple R	R^2^	Std Error	*p*-Value (X1)	Coef X1	F-Value (ANOVA)	*p*-Value (ANOVA)
SMC vs. EC 10 cm	0.942	**0.89**	1.718	**<0.001**	14.04	54.86	**<0.001**
SMC vs. EC 20 cm	0.939	**0.88**	1.922	**<0.001**	15.16	52.03	**<0.001**
SMC vs. EC 30 cm	0.916	**0.84**	2.676	**<0.001**	19.13	36.28	**<0.001**
SMC vs. EC 50 cm	0.123	0.02	3.705	0.75	−3.76	0.11	0.75
SMC vs. EC 70 cm	0.042	0.00	3.035	0.91	1.16	0.01	0.91

**Table 4:** Seasonal growth stages of *C. moschata* ‘Butternut’, with irrigation (mm), precipitation (mm), reference evapotranspiration (ET_o_, mm day^−1^), and crop evapotranspiration (ET_a,_ mm day^−1^) during the 2020 growing season.

CGS	Irrigation	Precipitation	ET_o_ (mm)		ET_a_ (mm)	
	(mm)	(mm)	Daily (mm day^−1^)	Period (mm)	Daily (mm day^−1^)	Period (mm)
I	n.d.	6.8	3.9	78.0	1.6	31.2
II	n.d.	1.0	4.8	143.8	3.3	98.4
III	n.d.	23.0	3.8	114.9	3.7	110.3
IV	n.d.	0	4.2	83.9	3.4	67.4
FCS	450 *	30.8	---	420.5	---	307.3

* Irrigation values per CGS were not recorded; total irrigation (450 mm) was applied throughout the crop season. (CGS = crop growth stage; FCS = full crop season; n.d. = no data per stage; daily ET_o_ and ET_a_ = average per period; period ET_o_ and ET_a_ = cumulative values).

**Table 5:** Statistical summary of the linear regression models relating FAO K_C_ to vegetation indices. Values in bold indicate statistically significant or relevant correlations, defined as R^2^ > 0.7 with *p* < 0.05, or an ANOVA *p*-value < 0.05 (*n* = 26).

Index	Multiple R	R^2^	Standard Error	*p*-Value (Coefficient X1)	Coefficient of X1	F-Value (ANOVA)	*p*-Value (ANOVA)
NDVI	0.857	**0.734**	0.112	**<0.001**	1.393	66.320	**<0.001**
SAVI	0.857	**0.735**	0.112	**<0.001**	0.930	66.542	**<0.001**
EVI	0.707	0.500	0.153	**<0.001**	0.405	24.012	**<0.001**
GCI	0.821	0.674	0.124	**<0.001**	0.245	49.577	**<0.001**

Note: Multiple R = Pearson’s correlation coefficient between observed and predicted *K_cb_* values; R^2^ = coefficient of determination; Standard Error = residual standard error of regression; Coefficient of X_1_ = slope of the regression line; F-value and *p*-value (ANOVA) indicate overall model significance. All the models used the intercept term.

**Table 6:** Linear regression R^2^ values between FAO-derived K_c_ and vegetation indices (VIs) across individual and combined crop growth stages (CGS). Values in bold indicate strong correlations (R^2^ > 0.7).

Index	I	II	III	IV	I + II	I + II + III	II + III	II + III + IV	III + IV
NDVI	N/A	0.692	N/A	**0.830**	**0.769**	**0.843**	**0.839**	0.472	**0.894**
SAVI	N/A	0.684	N/A	**0.863**	**0.767**	**0.842**	**0.842**	0.474	**0.901**
GCI	N/A	**0.702**	N/A	**0.780**	**0.816**	**0.848**	**0.739**	0.397	**0.787**
EVI	N/A	0.206	N/A	**0.784**	0.620	**0.700**	0.371	0.184	**0.789**

Note: N/A indicates insufficient data to compute reliable correlations.

**Table 7:** Regression equations between VIs and K_c_s including model fit parameters (R^2^, RMSE, and BIAS) for NDVI, SAVI, GCI, and EVI.

Vegetation Index	Regression Equation (K_c_ = a × VI + b)	R^2^	RMSE	BIAS
NDVI	K_c_ = 1.393 × NDVI − 0.042	0.734	0.089	0.011
SAVI	K_c_ = 0.930 × SAVI − 0.044	0.735	0.086	0.005
EVI	K_c_ = 0.405 × EVI + 0.278	0.500	0.132	0.073
GCI	K_c_ = 0.245 × GCI + 0.141	0.674	0.100	0.062

**Table 8:** Analysis of the variation in *C. moschata* ‘Butternut’ ET_a_ using the FAO-56 method and RS-assisted vegetation indices (NDVI, SAVI, EVI, and GCI), along with yield, water productivity and water use efficiency during the experimental season. Variations (Var.) in ET_a_ and WUE are calculated as (RS-A − FAO-56/FAO-56) × 100%.

Vegetation Index	ET_a_ FAO-56 (mm)	ET_a_ RS-A (mm)	Var. ET_a_ (%)	Y (t ha^−1^)	WP (kg m^−3^)	WUE FAO-56 (kg m^−3^)	WUE RS-A (kg m^−3^)	Var. WUE (%)
NDVI	307.3	312.6	2.9%	40	8.32	0.64	0.658	2.8%
SAVI	307.3	316.4	3.0%	40	8.32	0.64	0.658	2.8%
GCI	307.3	318.0	3.5%	40	8.32	0.64	0.661	3.3%
EVI	307.3	317.4	3.3%	40	8.32	0.64	0.660	3.2%

ET_a_—actual evapotranspiration (mm); Y—total *C. moschata* ‘Butternut’ production (t ha^−1^); WP—water productivity calculated as Y/(I + P), in kg m^−3^; WUE—water use efficiency calculated as ET_a_/(I + P), in kg m^−3^; I—irrigation; P—precipitation. Variations in ET_a_ and WUE (%) represent the relative difference between RS-A and FAO-56 estimates.

**Table 9:** Number of cloud-free Sentinel-2 scenes by phenological stage during the 2020 experimental cycle.

Crop Growth Stage	Date Range (2020)	Number of Images
I	6–25 June	4
II	26 June–25 July	7
III	26 July–24 August	7
IV	25 August–13 September	8
Total	---	26

## Data Availability

The data presented in this study are available upon request from the corresponding author.

## Abbreviations

The following abbreviations are used in this manuscript:

AWC	Available water capacity
AWS	Soil water storage
BIAS	Systematic deviation or bias of model
BOA	Bottom-of-atmosphere
B2	Sentinel-2 band 2
B3	Sentinel-2 band 3
B4	Sentinel-2 band 4
B8	Sentinel-2 band 8
C	Conductivity
Ca	Calcium
CBI	Centre for the Promotion of Imports from Developing Countries
CGS	Crop growth stage
EC	Electrical conductivity
ETa	Actual crop evapotranspiration
ETo	Reference evapotranspiration
EVI	Enhanced vegetation index
F-value	F-statistics for regression significance (ANOVA)
*fc*	Fraction of ground covered by vegetation
*few*	Exposed and wet fraction of the soil that is subject to evaporation
*fw*	Fraction of surface wetted by irrigation
FAO-56	Food and Agriculture Organization
FCS	Full crop season
G	Gain factor in EVI
GCI	Green chlorophyll index
GEE	Google Earth Engine
I	Irrigation
K	Potassium
K_c_	Crop coefficient
K_c_max_	Maximum crop coefficient
K_cb_	Basal crop coefficient
K_cb_end_	Late-season basal crop coefficient
K_cb_ini_	Initial stage basal crop coefficient
K_cb_mid_	Mid-season basal crop coefficient
K_e_	Soil evaporation coefficient
L_s_	Soil correction factor in SAVI
LVID	Lis Valley Irrigation District
Mg	Magnesium
N/A	Not available / insufficient data
NDVI	Normalized difference vegetation index
NIR	Near-infrared reflectance
P	Precipitation
*p*-value	Probability value for statistical tests
PSU	Practical Salinity Units
QGIS	Quantum Geographic Information System
R	Resistivity
R^2^	Coefficient of determination
RED	Red reflectance
RDO	Dissolved oxygen concentration
RHmean	Mean relative humidity
RMSE	Root mean square error
RS	Remote sensing
S	Salinity
SAVI	Soil adjusted vegetation index
SMC	Soil moisture content
SS	Suspended solid concentration
TDR	Time domain reflectometry
Tmean	Mean air temperature
Tmax	Maximum air temperature
u_2_	Wind speed at 2 m height
UAV	Unmanned aerial vehicle
VI	Vegetation index
WP	Water productivity
WUE	Water use efficiency
